# Research on Coal Dust Wettability Identification Based on GA–BP Model

**DOI:** 10.3390/ijerph20010624

**Published:** 2022-12-29

**Authors:** Haotian Zheng, Shulei Shi, Bingyou Jiang, Yuannan Zheng, Shanshan Li, Haoyu Wang

**Affiliations:** 1 Joint National-Local Engineering Research Centre for Safe and Precise Coal Mining, Anhui University of Science and Technology, Huainan 232001, China; 2 School of Safety Science and Engineering, Anhui University of Science and Technology, Huainan 232001, China; 3 Mining Enterprise Safety Management of Humanities and Social Science Key Research Base in Anhui Province, Anhui University of Science and Technology, Huainan 232001, China; 4 School of Economics and Management, Anhui University of Science and Technology, Huainan 232001, China; 5 Key Laboratory of Industrial Dust Prevention and Control & Occupational Health and Safety, Ministry of Education, Anhui University of Science and Technology, Huainan 232001, China

**Keywords:** coal dust wettability, BP neural network, GA–BP model, identification

## Abstract

Aiming at the problems of the influencing factors of coal mine dust wettability not being clear and the identification process being complicated, this study proposed a coal mine dust wettability identification method based on a back propagation (BP) neural network optimized by a genetic algorithm (GA). Firstly, 13 parameters of the physical and chemical properties of coal dust, which affect the wettability of coal dust, were determined, and on this basis, the initial weight and threshold of the BP neural network were optimized by combining the parallelism and robustness of the genetic algorithm, etc., and an adaptive GA–BP model, which could reasonably identify the wettability of coal dust was constructed. The extreme learning machine (ELM) algorithm is a single hidden layer neural network, and the training speed is faster than traditional neural networks. The particle swarm optimization (PSO) algorithm optimizes the weight and threshold of the ELM, so PSO–ELM could also realize the identification of coal dust wettability. The results showed that by comparing the four different models, the accuracy of coal dust wettability identification was ranked as GA–BP > PSO–ELM > ELM > BP. When the maximum iteration times and population size of the PSO algorithm and the GA algorithm were the same, the running time of the different models was also different, and the time consumption was ranked as ELM < BP < PSO–ELM < GA–BP. The GA–BP model had the highest discrimination accuracy for coal mine dust wettability with an accuracy of 96.6%. This study enriched the theory and method of coal mine dust wettability identification and has important significance for the efficient prevention and control of coal mine dust as well as occupational safety and health development.

## 1. Introduction

With the rapid development of the global economy and increasing energy consumption, the coal market is in short supply, the mechanization level of coal mining has been greatly improved, and the safe and efficient mining of coal continues to mature. However, the problem of the high concentration of dust, which has long affected the safety of coal mine production and threatened the occupational health of miners, is still very serious. Coal mine dust mainly comes from the mining area, driving area, shotcrete working area, transportation, and other links area, among which the dust production amount of the mining area accounts for more than 85% of the whole mine [1]. Taking the fully mechanized mining area as an example, under the conditions that no dust prevention measures are taken, the time-weighted total dust concentration in the main working area of the coal mine operation can reach 500–850 mg/m^3^, and the respirable dust concentration can reach 300–500 mg /m^3^ [2]. According to incomplete statistics, coal dust in more than half of coal mines is explosive [3]. When the suspended coal dust in coal mine reaches a certain mass concentration and there is enough of a heat source and enough oxygen, a coal dust explosion may occur. The damage degree of a coal dust explosion is much greater than that of a gas explosion, which can easily cause serious casualties and property losses. The statistics of coal dust explosion accidents with more than 100 deaths since the founding of New China are shown in Table 1.

**Figure 1:**
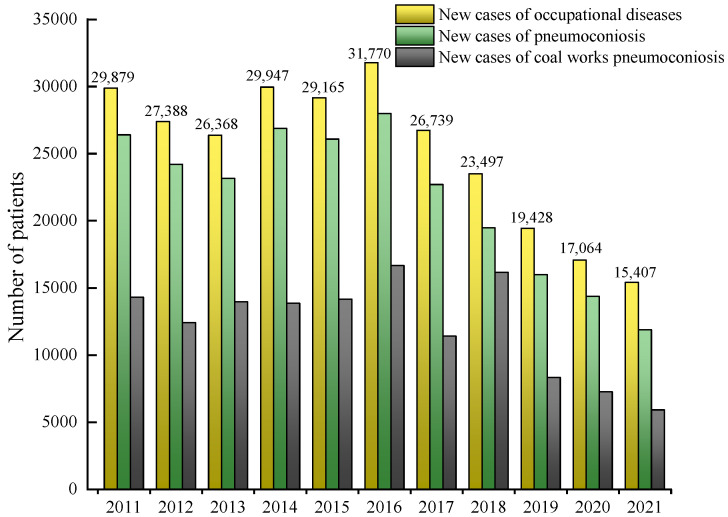
New cases of occupational diseases and pneumoconiosis in coal workers in the past 10 years.

The high concentration of dust in coal mines not only poses a serious threat to the safety of mine production but also causes lung, cardiovascular, and brain diseases in coal mine workers. China ranks first in the world in the number of new cases, cumulative cases, and deaths due to occupational diseases, mainly pneumoconiosis in coal workers. The statistics of new occupational disease cases and pneumoconiosis cases in coal workers in China in the past 10 years are shown in Figure 1. Since 2011, the death toll of coal mine accidents has decreased year by year, but the numbers of newly reported cases of pneumoconiosis in coal workers have increased year by year. From 2011 to 2021, about 276,000 new cases of pneumoconiosis were reported nationwide, among which about 134,000 cases of pneumoconiosis in coal workers were reported, accounting for 48.6 percent of the total. In 2018, the number of new cases of pneumoconiosis in coal workers accounted for 83 percent of the total number of cases of pneumoconiosis. According to incomplete statistics, about 1900 people die every year from pneumoconiosis in coal mines nationwide, which is six times as many as the death toll from coal mine disasters and other work-related accidents. The adverse effects of coal mine dust on the occupational health of coal mine workers have far exceeded that of safety accidents in coal mine production. Therefore, the research on coal mine dust prevention and control is not only conducive to preventing coal dust explosion accidents but also conducive to promoting the occupational safety and health development of coal mine employees.

At present, the commonly used dust prevention technologies in coal mines mainly include coal seam dust suppression by water injection [4,5,6], ventilation dust removal [7,8,9], foam dust removal [10,11], spray dust removal [12,13], air curtain dust control [14,15,16], dust removal by a dust collector [17,18,19], etc. Among them, coal dust wettability directly affects the effects of coal seam water dust suppression and spray dust removal. Quickly and accurately identifying the wettability of coal dust in different mining areas and adopting different effective prevention and control measures for coal dust with varying wettability have become the major demands in the field of coal mine safety production and occupational safety and health. Scholars at home and abroad have conducted in-depth research on the relationship between the physical and chemical properties of coal dust and its wettability. Generally speaking, increasing the moisture content is considered to improve the wettability of coal dust [20], but Hu [21] showed that the moisture content had little effect on the wettability. Increasing the fixed carbon content can make coal dust more hydrophobic [22], but Hu [21] showed that increasing the fixed carbon content can also make coal dust more hydrophilic. The effects of ash and volatile substances on the wettability of coal dust are also varied [21,22,23]. Cheng et al. [24,25] proposed that aromatic groups and hydroxyl groups were the two main factors affecting the wettability of coal dust, and the increase in the C-H bond content of aromatic hydrocarbons would lead to a smaller contact angle between the droplet and the coal dust surface. Gao et al. [26] revealed that the content of phenolic hydroxyl and fixed carbon determined the wettability. In addition, the mineral content in coal dust has been shown to have a significant impact on its wettability [27]. The quartz content, as a representative of the primary mineral in coal, is the main mineral factor affecting the wettability of coal dust [28]. The study of Zhao et al. [29] showed that with the increase in the quartz content, the wettability of coal dust first decreases and then increases. Based on the relationship between the functional group content and the contact angle, Zhen [30] found that the relationship between the content of cyclic hydroxyl groups and the size of the contact angle was the most significant. Wang [31] selected three different types of coal samples and eighteen different samples with six different particle sizes and found that the wettability of dust with the same properties decreased with the decrease in the particle size.

Many scholars have established the relationship between the wettability of coal dust and its physical and chemical properties through origin fitting or SPSS multiple regression analysis and have made certain research progress. However, the influence of the physical and chemical properties of coal dust on its wettability is still unclear. An intelligent algorithm is a kind of scientific method to simulate natural phenomena and processes, which is widely used to solve prediction and classification problems [32]. However, its application to the wettability identification of coal dust is rarely reported. Based on this, this study proposed a coal dust wettability discrimination method based on Genetic Algorithm (GA) to optimize the BP neural network. GA was used to optimize the initial weight and threshold of the BP neural network, and the optimized model was used to carry out data fusion on the physical and chemical properties of coal dust. By establishing a mathematical model to correlate the physical and chemical properties of coal dust with the wettability, the contact angle of coal dust could be predicted directly, and the wettability of coal dust could be identified quickly and accurately.

## 2. Influencing Factors and Data Acquisition of Coal Dust Wettability

### 2.1. Physical and Chemical Properties of Coal Dust Wettability

Coal is a complex sedimentary rock composed of organic and inorganic components [33]. Different types of coal are affected by different natural environmental conditions, and their physical and chemical properties are quite different. Coal dust wettability refers to the ability of coal dust to be wetted by liquid. Coal dust from different coal mines or different coal seams has different physical and chemical compositions, and these complex physical and chemical properties determine the varying wettability of coal dust [34].

The physical properties of coal mainly include moisture (M), ash (A), volatile (V), fixed carbon (Fc), and pore structure characteristics. The industrial analysis of coal refers to the general term for the determination of four analysis items including the moisture, ash, volatile, and fixed carbon of coal [35]. Studies have shown that the higher the moisture content in coal, the stronger the wettability of the coal dust [36]. The ash content of coal refers to the residue left after the complete combustion of the coal, which is also an important index affecting the wettability of coal dust [37,38]. The volatilization of coal refers to the content of moisture in the escaped material (gas or liquid) after the coal is isolated and heated by air at a certain temperature. The fixed carbon of coal is also an indicator of the metamorphism degree of coal as well as its volatile matter. The pore structure of coal mainly includes the specific pore volume and average pore diameter. The chemical properties of coal mainly include the content of C, H, O, N, S, and other elements. The elemental analysis of coal is a general term for the detection and analysis of the content of C, H, O, N, S, and other elements in coal [39]. The content of elements in coal also has an important effect on the wettability of coal dust. As the basic structural unit of coal is the aromatic ring system with carbon as the skeleton, there are side chains and functional groups composed of carbon, hydrogen, oxygen, and a small amount of nitrogen and sulfur atoms around the aromatic ring, such as carboxyl groups (-COOH), hydroxyl groups (-OH) and methoxy groups (-OCH3) [40]. This shows that the organic matter in coal is mainly composed of carbon, hydrogen, oxygen, nitrogen, sulfur, and other elements [41]. The degree of metamorphism of coal is different, its structural units are different, and the content of elements is also different. The carbon content increases with the increase in coal metamorphism, the hydrogen and oxygen content decrease with the increase in coal metamorphism, and nitrogen and sulfur have no relationship with the degree of metamorphism [42]. Therefore, the physical and chemical properties of coal together determine the wetting capacity of coal dust.

### 2.2. Methods for Measuring Wettability of Coal Dust

**Figure 2:**
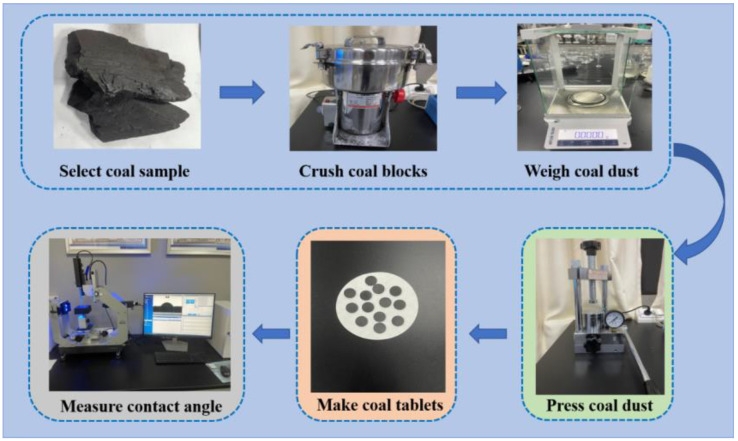
Schematic diagram of contact angle experimental procedure.

At present, coal dust wettability measurement methods mainly include the capillary rising method, the settlement rate method, the contact angle measurement method [43,44,45,46,47], etc. The capillary rise method involves the loading of dust into a bottomless glass test tube with filter paper at the bottom. The test tube is placed vertically on the immersion surface, and the end face of the bottom is placed in contact with the immersion surface. The height of dust infiltration of a group of a corresponding time is measured to represent the wettability of coal dust. The sedimentation rate method refers to putting coal dust per unit mass into a beaker filled with solution and observing the time when the dust is completely immersed in the solution so as to calculate the dust settlement rate in the solution. Its disadvantages are that it takes a long time and its efficiency is too low. The contact angle method is widely used to characterize the wettability of solid particles. Prior to the test, solid particles are pressed into sheets using a high-pressure die to create a smooth surface, and droplets are then suspended on the surface. The contact angle “θ” is measured using the light image of the droplet and the surface. The smaller the contact angle, the better the wettability of the solid particles [48]. The operation steps of coal dust wetting contact angle measurement are as follows: the coal is pulverized to a particle size less than 200 mesh, the surface moisture of coal sample is dried, a 0.2 g coal sample is taken and pressed with tablet press under 30 MPa pressure, and it is then pressed into forming body with a diameter of about 13 mm and a thickness of 2 mm. In a constant temperature room, manual sampling is performed followed by drip treatment using a contact angle measuring instrument, which is repeated three times to take the average value, as shown in Figure 2. The method of contact angle measurement has a certain objectivity and short time. It overcomes the disadvantages of strong subjectivity and low efficiency of the methods of capillary rising and settling rate, and its results are more real and reliable.

However, it can be seen from Figure 2 that contact angle measurement requires a large variety of experimental instruments and complicated operation procedures. The coal dust wetting contact angle model constructed by an intelligent algorithm can efficiently and accurately calculate the coal dust contact angle and thus determine the coal dust wettability. The application of an intelligent algorithm in determining the contact angle of coal dust wetting not only makes full use of the advantages of being relatively objective and providing accurate contact angle measurement results but also avoids the disadvantages of having a complicated contact angle measurement process. The influencing factors of coal dust wettability are complex and have high dimensional nonlinear characteristics. “High dimension” means that the wetting ability of coal dust is not determined by a single or a few factors but by the numerous physical and chemical properties of coal. A GA optimization BP neural network algorithm is a scientific calculation method, which is constantly searching and improving [49]. It makes full use of the learning ability of a BP neural network and the global search ability of a genetic algorithm to build a reasonable adaptive recognition model, which can be applied in the field of coal dust wettability recognition.

### 2.3. Data Acquisition

Based on the literature review, the authors obtained 35 sets of data on the basic physical and chemical properties and contact angles of coal dust [50]. The experimental coal samples were from Fuxin, Tieling, Shenyang, Inner Mongolia, Shandong, and Shanxi, including more than 10 kinds of coal samples with different metamorphic degrees such as lignite, long flame coal, gas coal, fat coal, lean coal, and anthracite. The physical and chemical characteristics of coal dust and the contact angle of coal dust were measured under laboratory conditions. On this basis, a contact angle estimation model with the physical and chemical characteristics of coal dust as the input parameters and the contact angle of coal dust as the output parameters was constructed. Among them, the physical and chemical characteristics of coal dust mainly included 13 indicators, namely moisture (Aad) (%), ash (Vad) (%), volatile (Mad) (%), fixed carbon (FCad) (%), carbon content (%), hydrogen content (%), oxygen content (%), sulfur content (%), nitrogen content (%), the molar ratio of hydrogen to carbon, the molar ratio of oxygen to carbon, the specific pore volume (cm^3^.g^−1^), and the average pore diameter (Å), which were recorded as X1-X13, respectively. The physical and chemical property data of the 35 groups of coal dust are shown in Table 2.

## 3. Coal Dust Wettability Prediction Model Based on GA–BP

### 3.1. GA–BP Algorithm

**Figure 3:**
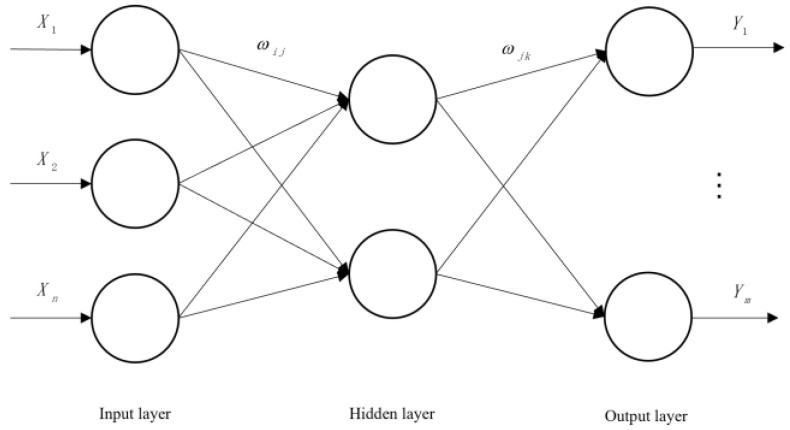
Topology diagram of BP neural network.

The BP neural network is a multi-layer feedforward neural network, which is mainly characterized by forward signal transmission and back error propagation [51]. In forward propagation, the input layer is processed layer by layer from the input layer through the hidden layer until the output layer. The state of the neurons in each layer only affects the state of the neurons in the next layer. If the output layer cannot obtain the expected output, it is transferred to the back propagation, and the network weight and threshold are adjusted according to the prediction error so that the predicted output of the BP neural network is constantly approaching the expected output. Setting X1, X2..., Xn as the input values of the BP neural network, Y1, Y2..., Ym are the predicted values of the BP neural network, and Wij and Wjk are the weights of the BP neural network. The BP neural network can be regarded as a nonlinear function. The input and predicted values of the network are the independent and dependent variables of the function, respectively. When the number of input nodes is *n* and the number of output nodes is m, the BP neural network expresses the function mapping relationship from *n* independent variables to m dependent variables. The topology structure of BP neural network is shown in Figure 3.

A genetic algorithm is a computational model of the biological evolution process simulating natural selection and the genetic mechanism of Darwinian biological evolution, and it is a method used to search for an optimal solution by simulating the natural evolution process [52]. It was first proposed by Professor J. Halland from the University of Michigan in the United States. It simulates the phenomena of replication, crossover, and variation occurring in natural selection and heredity. Starting from any initial population, it generates a group of individuals better adapted to the environment through random selection, crossover, and mutation operations. The population is made to evolve to better and better areas in the search space so that there is generation after generation of continuous evolution, and finally a group of individuals most adapted to the environment is converged to in order to obtain the optimal solution to the problem.

Previous studies have shown that in the process of gradient descent optimization, a BP neural network has defects such as slow learning convergence speed, the network structure being difficult to determine, and a local minimum value being easy to fall into in network training, resulting in a decline in application value [53]. A genetic algorithm is an adaptive global search optimal solution algorithm formed in the process of simulating natural genetic mechanisms and evolution. It has the advantages of parallelism, robustness, and global optimality. It can effectively resolve the risk that the training process of a BP neural network becomes limited to the local optimal and optimize the initial weight and threshold of a BP neural network to improve the stability of the BP neural network [54]. In this study, GA was combined with a BP neural network, and the initial weight and threshold of the BP neural network were optimized by GA to solve the defects of the BP neural network, such as it being too dependent on experience and having a slow convergence speed, and this was applied to the wettability identification of coal dust.

**Figure 4:**
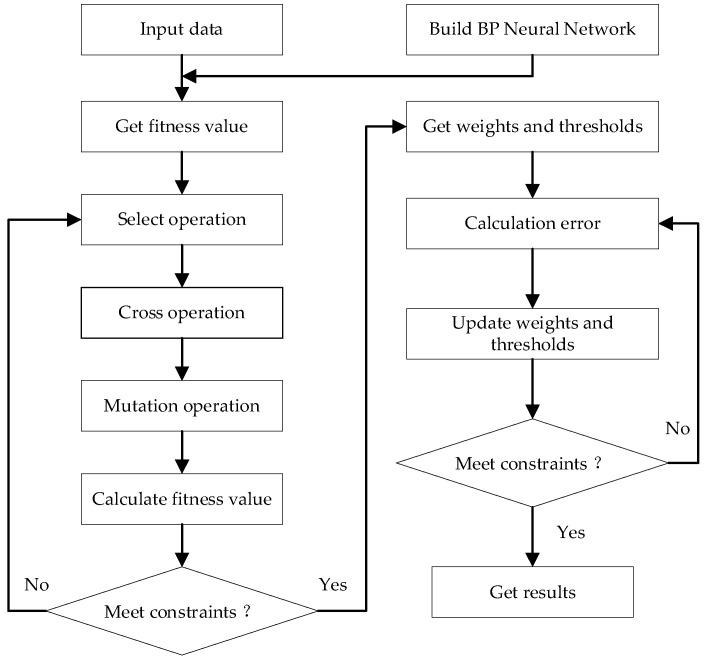
Flow chart of GA-optimized BP neural network.

The genetic algorithm optimization of the BP neural network was based on the establishment of a BP neural network structure using a genetic algorithm to optimize the weight and threshold of the neural network, and then the optimized BP neural network was used for analysis and prediction. The training flow chart is shown in Figure 4.

The process of optimizing the BP neural network based on the genetic algorithm is as follows:

(1) Read data.

(2) Preprocess data as follows:

(1) $$x_{k}=\frac{x_{k}-x_{min}}{x_{max}-x_{min}}$$

where $x_{min}$ is the lowest value of a data sequence and $x_{max}$ is the highest value of the data sequence.

(3) Set the optimal hidden layer node number and select an empirical formula as follows:

(2) $$l<\sqrt{\left(m+n\right)}+a$$

where l, m, and n are the numbers of the hidden layer, input layer, and output layer nodes, respectively, and the value of a is typically between 0 and 10.

(4) Perform the initialization, selection, crossover, and mutation of the GA.

The GA used in this study adopted the roulette method for the selection operation, and the selection probability $P_{i}$ of individual i is expressed as

(3) $$f_{i}=k/F_{i}$$

(4) $$P_{i}=\frac{f_{i}}{\sum_{j=1}^{N}f_{i}}$$

where $F_{i}$ is the individual fitness, k is the coefficient, and N is the number of individuals in the population.

Real number coding and crossover processing are performed on the individual, and the crossover operation method of the k chromosome, $a_{k}$, and l chromosome, $a_{l}$, at position j is as follows:

(5) $$a_{kj}=a_{kj}\left(1-b\right)+a_{lj}*b$$

(6) $$a_{lj}=a_{lj}\left(1-b\right)+a_{kj}*b$$

where b is a random number in the interval [0, 1].

The mutation operation on gene j of individual i is as follows:

(7) $$a_{ij}=\left\{\begin{array}{c} a_{ij}+\left(a_{ij}-a_{max}\right)*f\left(g\right),\;\;\;\;\;r>0.5\\ a_{ij}+\left(a_{min}-a_{ij}\right)*f\left(g\right),\;\;\;\;\;r\leq 0.5 \end{array}\right.$$

where $a_{max}$ is the upper bound of $a_{ij}$, $a_{min}$ is the lower bound of $a_{ij}$, fg=(1−gGmax)2, $r_{2}$ is a random evolutionary number, g is the current number of iterations, $G_{max}$ is the maximum number of evolutions, and r is a random number in [0, 1].

(5) Perform real number coding on the population, where the fitness value, F, is the error between the predicted and expected data, expressed as follows:

(8) $$F=k\left(\overset{n}{\underset{i=1}{\sum }}abs\left(y_{i}-o_{i}\right)\right)$$

where n is the number of output nodes of the BP neural network, $y_{i}$ is the expected output value of node i of the BP neural network, $o_{i}$ is the predicted output value for node i, and k is a coefficient.

(6) Circularly iterate the optimal initial weight value and threshold value.

(7) Construct a BP neural network using the optimal initial weight value and threshold value obtained via circular iteration.

(8) Import training data, input_train, to train a BP neural network.

(9) Transfer the test data, input_test, in the dataset into the trained neural network for testing and perform inverse normalization processing on the prediction data.

(10) Perform an error analysis on the expected and predicted values.

### 3.2. Establishment of Model

This study was based on the MATLAB R2020a software for experimental research. The training set and testing set were randomly selected, among which thirty sets of training and five sets of testing were used for this model. In the MATLAB neural network toolbox, the newff () function was used to create the BP neural network, the mapminmax () function was used to normalize and de-normalize the data, the train () function was used to train, and the sim () function was used to complete the network simulation. This model was based on the rand () function in MATLAB, which randomly selected thirty samples out of the thirty-five as the training set and the remaining five samples as the test set. The purpose of the training set was to build the model, while the purpose of the validation set was to use each model to record the accuracy of the model in order to find out the model with the best effect. The validation set was automatically generated by newff () function in the MATLAB neural network toolbox to select the parameters corresponding to the model with the best effect, that is, to adjust the model parameters. After obtaining the optimal model through the training set and validation set, the test set was used for the model prediction.

#### 3.2.1. GA–BP Hidden Layer Number Selection

According to Formula (2), the number of hidden layers in this test was between three and fifteen. There was an inverse relationship between the RRMSE value and the model fitting effect, that is, the smaller the RRMSE value, the better the fitting effect. The training process showed that when the number of hidden layer nodes was three, the corresponding root mean square error reached the minimum of 0.016256, so the number of hidden layer nodes in the neural network was determined to be three. The corresponding relationship between the number of hidden layer nodes and the root mean square error is shown in Table 3.

In this study, 13 influencing factors of coal dust wettability were taken as the input parameters, and the estimated value of the contact angle was taken as the output parameter. Therefore, the input and output parameters of the fitting nonlinear function in the model were thirteen and one, respectively. The structure of the GA–BP model was 13–3–1, namely, there were thirteen nodes in the input layer, three nodes in the hidden layer, and one node in the output layer. There were forty-two common weights (13 × 3 + 3 × 1 = 42) and four threshold values (3 + 1 = 4), so the individual coding length of the neural network was forty-six (42 + 4 = 46).

#### 3.2.2. Parameter Setting of GA–BP Model

**Figure 5:**
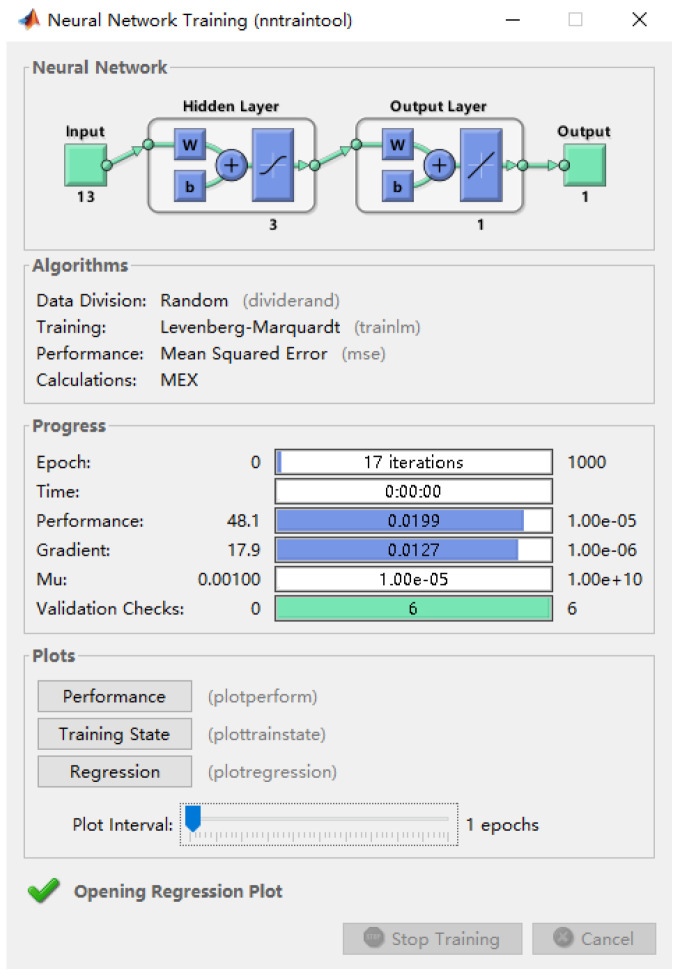
GA–BP network training parameters.

Goal represents the minimum error of the training goal; epochs represents the number of training sessions; show represents the display frequency of training iterations; lr represents the learning rate; mc represents the momentum factor; min_grad represents the minimum performance gradient; and max_fail represents the highest number of failures. These were set to 0.00001, 1000, 25, 0.01, 0.01, 10^−6^, and 6, respectively, in sequence. The common parameters of the BP neural network and GA–BP, including the minimum error of the training target, training times, learning rate, momentum factor, minimum performance gradient, and maximum failure times, were set as the same. The parameter settings of the GA–BP model are shown in Table 4, and the diagram of the network training parameters is shown in Figure 5.

## 4. Simulation Results and Performance Analysis

### 4.1. Simulation Results

**Figure 6:**
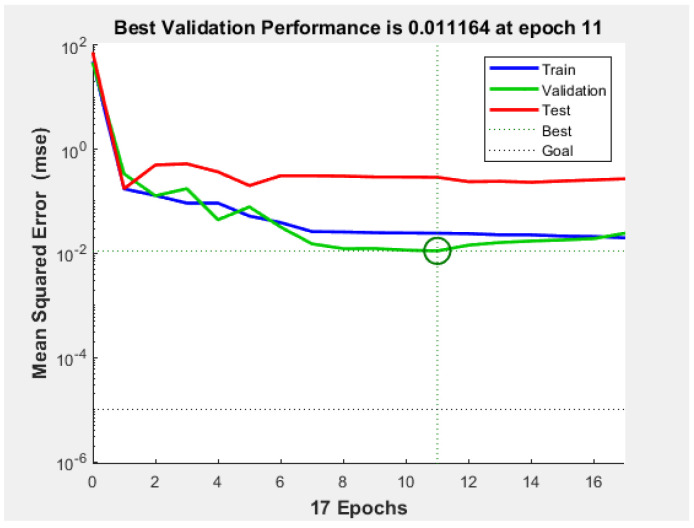
Mean square error of training set, verification set and test set.

This study was based on the MATLAB R2020a platform for the results simulation. First, the data was read, and then the BP neural network was optimized by the genetic algorithm to establish the prediction model. Figure 6 shows the mean square error of the training set, validation set, and test set. In the 11th generation, the root mean square error of the verification set reached the minimum value of 0.011164.

**Figure 7:**
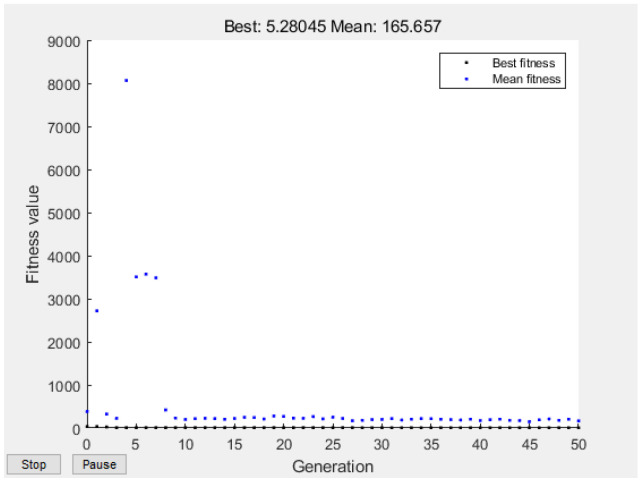
Genetic algorithm optimization process diagram.

**Figure 8:**
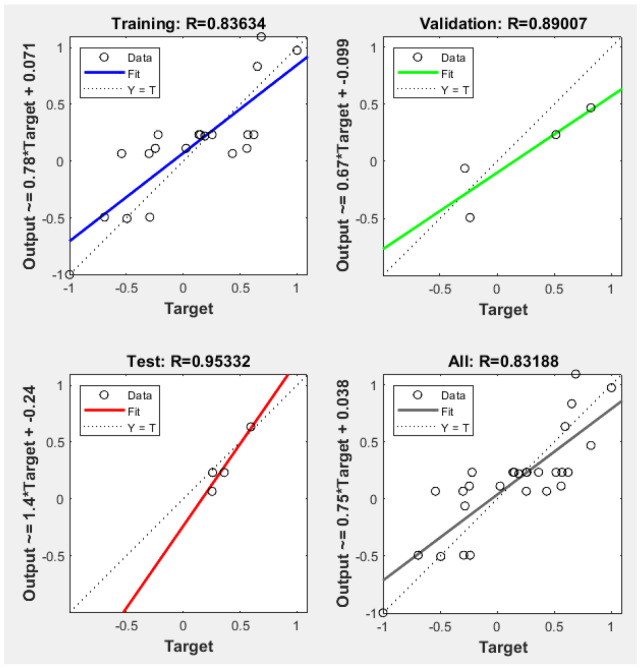
GA–BP linear regression results.

The optimization process of the genetic algorithm is shown in Figure 7. When the initial population size of the GA–BP model was 30 the maximum evolutionary algebra was 50, the cross probability was 0.8, the mutation probability was 0.2, the mean fitness of the population was 165.657, and the best fitness was reached in the 45th generation with an optimal fitness value of 5.28045. The GA–BP linear regression results are shown in Figure 8. The regression results (Figure 8) showed that the regression coefficient of the training set was 0.83634, that of the verification set was 0.89007, that of the test set was 0.95332, and that of the sample population was 0.83188. It showed that the fitting results of the overall data of the sample were ideal and the model was effective, which can be used for further analysis.

**Figure 9:**
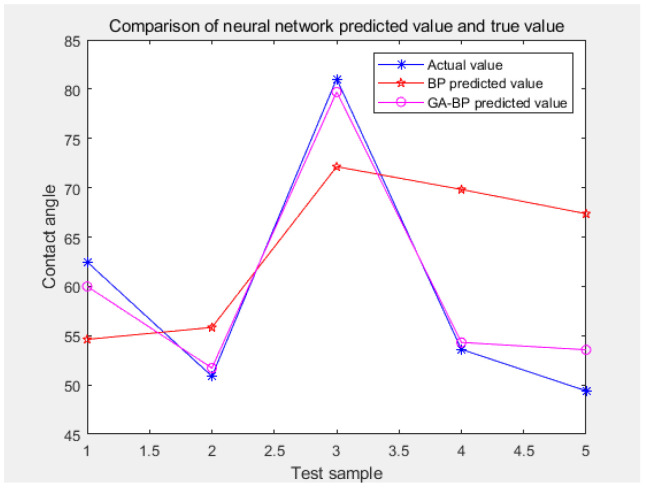
Simulation results of BP model and GA–BP model.

With the same parameter settings of the BP model and the GA–BP model, thirty-five groups of data were applied to the two models, respectively, and analyzed. The operation results showed that the contact angle values of the five randomly selected test sets were 62.462°, 50.87°, 81.031°, 53.6°, and 49.4°, respectively. The contact angle values estimated by the BP model were 54.61974°, 55.83009°, 72.13396°, 69.82025°, and 67.35999°, respectively. The contact angle values estimated by the GA–BP model were 59.9874°, 51.70715°, 79.70484°, 54.31024°, and 53.56109°, respectively. The BP neural network took 0.886169 s and the GA–BP model took 25.102379 s, as shown in Table 5. The simulation results diagram is shown in Figure 9.

### 4.2. Performance Analysis of Prediction Results of Different Algorithms

In order to evaluate the merits of the simulation results, this study selected the mean absolute percentage error (*MAPE*), root mean square error (*RMSE*), and mean absolute error (*MAE*) as the prediction evaluation indexes, where the calculation formulae are as follows:

(9) $$MAPE=\frac{1}{M}\overset{M}{\underset{i=1}{\sum }}\left|\frac{y_{i}-{\hat{y}}_{i}}{y_{i}}\right|\times 100\%$$

(10) $$RMSE=\sqrt{\frac{1}{M}\overset{M}{\underset{i=1}{\sum }}{\left(y_{i}-{\hat{y}}_{i}\right)}^{2}}$$

(11) $$MAE=\frac{1}{M}\overset{M}{\underset{i=1}{\sum }}\left|y_{i}-{\hat{y}}_{i}\right|\times 100\%$$

where $y_{i}$ is the actual value of the contact angle, $\hat{y_{i}}$ is the predicted value of contact angle, and M is the number of test samples.

As can be seen from the three fitting curves in Figure 6, both the BP neural network and the BP neural network optimized by the genetic algorithm had similar fitting trends between the predicted output and the actual output, but the BP neural network optimized by the genetic algorithm was more accurate than the standard BP neural network. The calculation results showed that the average absolute error of the standard BP neural network was 19.98%, and the recognition accuracy was 80.02%. The average absolute error of the GA-optimized BP neural network was 3.4%, and the recognition accuracy was 96.6%. By comparing the standard BP model with the GA–BP model, it was found that the GA–BP model was feasible in the discrimination of coal dust wettability and could significantly improve the accuracy of coal dust recognition.

**Figure 10:**
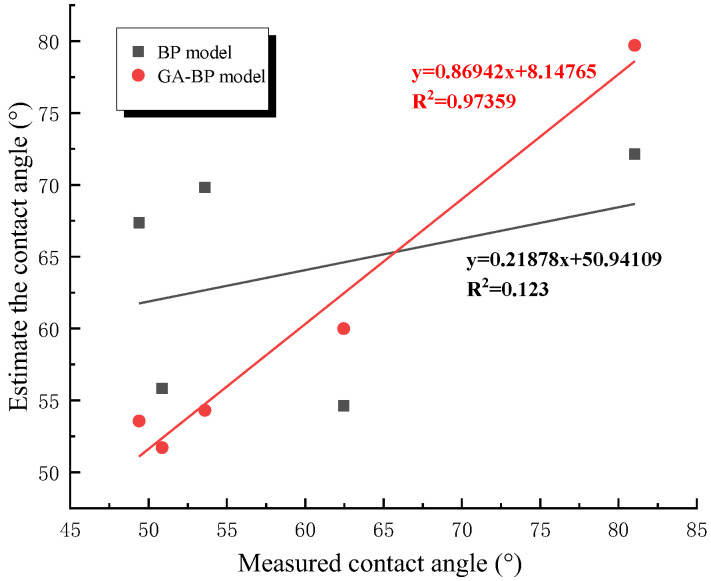
Correlation between measured value and estimated value of coal dust contact angle.

Through the correlation analysis between the measured value and the estimated value of the coal dust wetting contact angle, the merits and disadvantages of the model were further compared, and the results are shown in Figure 10. The GA–BP linear regression equation was y = 0.86942x + 8.14765, and the correlation coefficient was R^2^ = 0.97359. The BP linear regression equation was y = 0.21878x + 50.94109, and the correlation coefficient was R^2^ = 0.123. By comparison, it can be seen that the estimation results of the coal dust contact angle based on the standard BP neural network established by coal quality parameters were not accurate enough. The correlation coefficient of the coal dust contact angle estimation by the GA–BP model was large, the fitting equation was ideal, and the coal dust wettability identification accuracy was higher.

For further comparative study, the authors introduced the extreme learning machine algorithm (ELM) and the particle swarm optimization extreme learning machine algorithm (PSO–ELM) and applied them in the field of coal dust wetting identification. The ELM algorithm is a new type of feedforward neural network with a single hidden layer. It has the advantages of having a strong generalization ability and a fast learning speed and overcomes the shortcomings of traditional neural network learning algorithms, which require constant iteration, many training repetitions, a low learning efficiency, and a slow convergence speed. The PSO algorithm is a guided intelligent algorithm that mimics bird foraging. Compared to other optimization algorithms, the PSO algorithm has the advantages of a simpler definition, a higher computational efficiency, and easier operation.

The performance comparison of the different models is shown in Table 6. The comparison showed that the GA–BP model had the highest prediction accuracy in terms of RMSE, MAE, and MAPE, which was superior to the BP, ELM, and PSO–ELM models. Secondly, the prediction accuracy of the PSO–ELM model was better than that of the BP and ELM models. The BP model had the worst prediction accuracy. It can be seen that, compared with the other intelligent algorithms, the coal dust contact angle estimation model conducted through GA to optimize the weights and thresholds of the BP neural network had a higher prediction accuracy. By comparing the iteration time of the PSO algorithm and the GA algorithm, it was found that under the conditions that the maximum number iteration times (50 times) and the population size (20 generations) of the PSO algorithm and GA algorithm were the same, the PSO–ELM model took 3.09064 s and the GA–BP model took 25.102379 s, indicating that the PSO algorithm was superior to the genetic algorithm in terms of optimization speed. However, the genetic algorithm’s performance was due to the particle swarm optimization algorithm in terms of the optimization effort.

## 5. Conclusions and Discussion

(1) With the thirteen indicators of the physical and chemical properties of coal dust as the input parameters and the coal dust contact angle as the output parameter, a three-layer BP neural network for estimating the wetting contact angle of coal dust was constructed by using a logarithmic sigmoid-type function. The number of hidden layer neurons had a great impact on the neural network estimation results. Choosing the appropriate number of hidden layer neurons is beneficial for reducing the network estimation error. When the number of hidden layer neurons was three, the correlation coefficient between the estimated value of the coal dust wet contact angle and the measured value was R^2^ = 0.97359, the average absolute error was 3.4%, and the recognition accuracy was as high as 96.6%.

(2) In terms of RMSE, MAE, and MAPE, the prediction accuracy of the different models was different, and the order was GA–BP > PSO–ELM > ELM > BP. When the maximum number of iteration times and population size of the PSO algorithm and GA algorithm were the same, the running times of the different models were also different, and the order was ELM < BP < PSO–ELM < GA–BP. It can be seen that, compared with the other intelligent algorithms, the coal dust contact angle estimation model through GA used to optimize the weights and thresholds of the BP neural network had a higher prediction accuracy.

(3) This study innovatively proposed a systematic, scientific, and practical coal dust wettability contact angle estimation model (GA–BP model) to achieve the effective identification of coal dust wettability. The application of the GA–BP model in the prediction of the contact angle of coal dust wetting not only made full use of the advantages of having relatively objective and accurate contact angle measurement results but also effectively solved the disadvantages of requiring a complicated contact angle measurement process. The model is helpful for guiding the selection of dust control measures in coal mines and providing a guarantee for the safety of coal mine production and miners’ occupational safety and health.

## 6. Contributions and Limitations

A large number of dust particles are produced in the coal mining, driving, transportation, and reprinting processes of coal production. Because the dust particles cannot settle in time, coal dust pollution in underground working spaces is serious. At present, coal mines commonly use dustproof technology. First, before coal mining, a water injection process is used to improve the wettability of coal or reasonable mining technology is adopted to reduce the possibility of coal dust production. Second, specific dust control technology is used during mining to control the dust source so that the dust exists in a specific space and position and cannot be further diffused. Third, relevant dust removal technology or equipment are used, which enable the timely filtering or elimination of the dust generated. These methods have improved downhole conditions to some extent, but dust concentrations are generally still far above the national limits.

Due to the varying wettability of coal mine dust, the dust removal methods for coal dusts with different wettabilities are also different. Therefore, how to accurately and efficiently identify the wettability of coal mine dust is very important. Aiming at the problems of the influencing factors of coal mine dust wettability not being clear and the identification process being complicated, this study proposed a coal mine dust wettability identification method based on a genetic algorithm (GA) to optimize a BP neural network. Firstly, 13 indicators affecting the wettability of coal dust were determined, and then a GA–BP model was introduced to estimate the wettability contact angle of coal dust. The model took the chemical composition and structural parameters of coal quality in coal mines as the training samples, selected different excitation functions and the number of hidden layer neurons, and calculated the mean and standard deviation of the actual and expected output of the network model. The “appropriate” number of hidden layer neurons was determined; the optimal excitation function was determined by comparing the model running rate and output relative error under different excitation function conditions. The influence factors input into the coal dust wetting contact angle estimation model were optimized to determine a practical and effective contact angle estimation model. Finally, the standard BP model and the GA–BP model were compared, and the results showed that the GA–BP model had the highest prediction accuracy. By constructing a systematic, scientific, and practical model of coal dust wettability identification, the effective judgment of coal dust wettability was realized. This study enriched the theory and methods of coal mine dust wettability identification and has important significance for the efficient prevention and control of coal mine dust and occupational safety and health development. In addition, it not only provided a new idea for the scientific identification of coal dust wettability but also provided an important reference for scientific evaluation and prediction in other high-dimensional and nonlinear fields. Compared with the BP model, ELM model, and PSO–ELM model, the GA–BP model took a longer time. The BP neural network searched for feedback in turn according to the concept of a gradient, which requires a huge amount of computation. When further adding the GA algorithm, it needed to constantly select, cross, and mutate among the training data, which consumed more time. In addition, the larger initial population size and more maximum evolutionary algebra were the main reasons that affected the longer time consumption of the GA–BP model. In the future, we can improve the structure of neural network, reduce the population size, and reduce the amount of evolutionary algebra so as to ensure the high accuracy of the model and shorten the consumption time of the model as much as possible.

However, only 13 physical and chemical properties affecting the wettability of coal dust were considered in this study. In the next step, the types and contents of the functional groups of coal dust will be taken into account to comprehensively consider the factors affecting the wettability of coal dust. In addition, the data set in this study was small, and the next step is to collect more experimental data and continuously optimize the model so as to further improve the accuracy of coal dust wetting identification.

## Figures and Tables

**Table 1:** Statistics of coal dust explosion accidents with more than 100 deaths since the founding of New China.

Number	Data	Dust Explosion Accident Place	Death Toll
1	27 February 1950	Yiluo Mine of Xinyu Company, Henan Province	174
2	9 May 1960	Laobaidong Mine of Datong Company, Shannxi Province	684
3	28 November 1960	Longshanmiao Mine of Pingdingshan Company, Henan Province	187
4	24 December 1981	Minmetals of Pingdingshan Mining Company, Henan Province	133
5	21 April 1991	Sanjiaohe Mine of Hongdong Company, Shanxi Province	147
6	27 September 2000	Muchonggou Mine of Shuicheng Company, Guizhou Province	162
7	27 November 2005	Dongfeng Mine of Qitaihe Company, Heilongjiang Province	171

**Table 2:** Data of physical and chemical properties of 35 groups of coal mine dust.

Number	X1	X2	X3	X4	X5	X6	X7	X8	X9	X10	X11	X12	X13	Contact Angle (°)
1	32.74	13.73	12.42	41.11	76.39	5.25	13.52	0.95	0.89	0.82	0.13	0.01	347.20	48.69
2	18.14	11.59	0.69	69.58	88.38	3.80	3.46	3.13	1.24	0.52	0.03	0.01	298.60	81.46
3	6.34	34.82	4.24	54.60	78.83	5.28	13.57	0.95	1.37	0.80	0.13	0.01	434.40	78.50
4	19.62	13.05	2.35	64.98	89.41	3.28	5.37	1.01	0.93	0.44	0.05	0.01	296.00	73.77
5	17.32	29.50	2.86	50.32	81.35	5.13	11.37	1.27	0.88	0.76	0.10	0.01	402.40	63.87
6	20.58	7.12	3.76	68.54	86.23	3.62	7.19	1.78	1.18	0.50	0.06	0.02	269.40	82.68
7	6.75	31.27	8.46	53.52	77.39	5.11	15.78	0.67	1.05	0.79	0.15	0.01	300.80	50.87
8	34.71	21.91	3.58	39.80	72.03	7.73	17.63	1.68	0.93	1.29	0.18	0.01	358.40	48.31
9	16.93	12.55	1.63	68.89	88.01	4.36	3.65	2.85	1.13	0.59	0.03	0.01	253.00	93.66
10	24.75	25.73	7.42	42.10	75.56	5.08	16.91	0.93	1.52	0.81	0.17	0.01	236.80	67.54
11	2.47	39.71	4.08	53.74	81.84	5.38	10.56	0.54	1.68	0.79	0.10	0.01	356.60	76.64
12	23.45	24.51	3.87	48.17	84.75	4.25	8.70	1.10	1.20	0.60	0.08	0.01	148.40	65.24
13	6.49	35.83	4.99	53.69	79.96	5.02	13.28	0.66	1.08	0.75	0.12	0.01	134.40	63.45
14	20.08	18.48	8.35	53.09	77.02	5.73	14.37	0.81	2.07	0.89	0.14	0.01	218.40	59.47
15	13.99	35.71	2.67	47.63	81.63	5.54	9.75	1.13	1.95	0.81	0.09	0.01	176.70	67.70
16	64.38	17.47	5.62	12.53	44.55	5.07	47.22	1.83	1.33	1.37	0.79	0.03	134.60	23.57
17	28.33	28.12	10.37	33.18	71.15	4.83	21.73	1.24	1.05	0.81	0.23	0.02	148.40	39.64
18	37.41	17.87	6.51	38.22	78.32	6.62	12.81	1.22	1.03	1.01	0.12	0.01	305.60	50.28
19	15.88	21.56	2.58	59.98	81.73	4.16	10.08	2.42	1.61	0.61	0.09	0.01	221.80	79.45
20	10.52	32.64	4.27	52.57	81.28	5.38	11.36	0.74	1.24	0.79	0.10	0.01	353.60	71.26
21	15.39	28.55	15.74	40.32	73.34	3.04	21.08	1.05	1.49	0.50	0.22	0.01	420.80	50.02
22	40.60	20.04	9.47	29.89	66.87	7.59	20.75	1.84	0.95	1.36	0.23	0.03	136.80	34.33
23	16.04	10.13	3.98	69.85	82.67	4.23	9.52	2.04	1.54	0.61	0.09	0.01	277.00	87.34
24	24.24	30.42	4.39	40.95	77.54	5.26	15.32	0.69	1.19	0.81	0.15	0.01	157.00	41.28
25	14.63	16.58	8.56	60.23	80.09	4.46	13.63	0.95	1.87	0.67	0.13	0.01	356.20	78.21
26	8.42	38.06	2.57	50.95	84.93	4.92	6.83	2.09	1.23	0.70	0.06	0.01	375.80	80.35
27	25.85	18.58	12.52	43.05	72.14	4.55	21.42	0.83	1.06	0.76	0.22	0.01	241.20	48.10
28	30.76	12.34	1.57	55.33	89.75	4.77	3.27	0.82	1.39	0.64	0.03	0.01	252.60	67.53
29	27.32	17.64	9.09	45.95	88.32	4.94	4.67	0.94	1.13	0.67	0.04	0.01	216.80	76.04
30	32.96	18.29	5.83	42.92	78.78	4.86	13.35	1.28	0.73	0.74	0.13	0.01	188.60	60.56
31	8.44	33.52	5.36	52.68	79.13	4.91	14.21	0.83	0.92	0.74	0.13	0.01	155.20	62.46
32	34.06	19.25	6.68	40.00	78.95	5.96	12.94	1.65	1.50	0.91	0.12	0.01	364.40	50.87
33	28.37	26.74	2.84	42.05	80.85	5.37	10.46	2.04	1.28	0.80	0.10	0.01	249.60	81.03
34	17.87	20.52	17.77	43.84	72.80	5.14	18.68	1.75	1.63	0.85	0.19	0.01	306.60	53.60
35	28.39	20.32	10.56	40.73	68.37	5.02	23.58	1.77	1.26	0.88	0.26	0.01	345.80	49.40

**Table 3:** Root mean square error of node layer number model.

Layer	R_RMSE_
3	0.016256
4	0.047823
5	0.28504
6	0.067797
7	0.26669
8	0.18253
9	0.1357
10	0.0.1968
11	0.12199
12	0.028462
13	0.134831
14	0.0884
15	0.034375

**Table 4:** Parameter setting of BP and GA–BP model.

Parameter Setting	Value
Training goal minimum error (goal)	0.00001
Training times (epochs)	1000
Learning rate (lr)	0.01
Momentum factor (mc)	0.01
Minimum performance gradient (min_grad)	10^−6^
Maximum number of failures (max_fail)	6
Training iteration display frequency (show)	25
Activation function	Sigmoid

**Table 5:** Coal dust contact angle of different models.

Test Set	1	2	3	4	5
Contact angle measurement	62.462°	50.87°	81.031°	53.6°	49.4°
BP predicted value	54.61974°	55.83009°	72.13396°	69.82025°	67.35999°
GA–BP predicted value	59.9874°	51.70715°	79.70484°	54.31024°	53.56109°

**Table 6:** Performance comparison of different models.

Model	BP	ELM	PSO–ELM	GA–BP
RMSE/%	12.2549	8.4900	6.5839	2.2979
MAE/%	11.1759	7.3872	4.9410	1.9018
MAPE/%	0.1998	0.1188	0.0838	0.034
Time/s	0.886169	0.066717	3.090464	25.102379

## Data Availability

Not applicable.
